# Supplementary material for: Profiling of primary and phytonutrients in edible mahlab cherry (Prunus mahaleb L.) seeds in the context of its different cultivars and roasting as analyzed using molecular networking and chemometric tools
Source: PeerJ. 2023 Aug 30;11:e15908. doi: 10.7717/peerj.15908 (PMC10474835; doi:10.7717/peerj.15908)
Supplement: Supplemental Information 1 [file peerj-11-15908-s001.docx]

**Extraction procedure and sample preparation for UPLC-MS analysis**

Seeds were first lyophilized using Benchtop Freeze Dryer, LYO60B-1PT till complete dryness and powdered using Kenwood KHH326BK Multione Mixer 1000 W. Powdered seeds 20 mg of each plant was extracted using 1.5 ml 100% methanol (HPLC grade) with sonication for 15 min. The extracts were centrifuged at 12000 g for 20 min to get rid of any debris followed by filtration through a 22 µm pore size filter (Agilent, USA). Lyophilized dried seed powder were ground using pestle and mortar under liquid nitrogen. 30 mg of the obtained powder was then homogenized with 2.5 mL 100% MeOH HPLC grade (Sigma, Aldrich) containing 10 µg/mL umbelliferone (an internal standard for relative quantification) using sonication for 15 min. The extracts were vortexed vigorously and centrifuged at 12000 g for 20 min to get rid of plant debris followed by filtration through a 22 um pore size filter (Agilent, USA). A blank of 100% methanol containing 10 µg umbelliferone is included.

**UPLC-MS based multivariate data analysis and molecular networking**

Full scan /automated MSMS in data dependent mode was acquired in both negative and positive modes for all files (100- 1400 *m/z*) using a UPLC system coupled online with a 6540 UHD Accurate-Mass Q-TOF (Agilent Technologies) following exact conditions described in (Sallam et al. 2022). The raw files of UPLC-MS of all samples were converted into “.abf” files using ABF converter (https://www.reifycs.com/AbfConverter/). Accordingly, MS dial software (http://prime.psc.riken.jp/compms/msdial/main.html) was employed for data extraction with the following parameters: retention time (0-35 min), mass range (50-1000 Da), and accurate mass tolerance for MS1 (0.01 Da), and MS2 (0.025 Da) following exact procedure described in (Saied and Farag 2023). Peak area mass list was used and exported for multivariate data analysis. Positive and negative mode were not merged together but each modelled separately as depicted in Fig. 2. Umbelliferone spiked in samples as internal standard served only as anchor for alignment and further to confirm accuracy of MS spectrometer in formula prediction, and its reproducibility.

The exported data was subjected to unsupervised and supervised multivariate data analyses, *i.e.*: PCA, hierarchical clustering analysis (HCA), and OPLS-DA using SIMCA 14.1 software (Umetrics, Sweden) to show the segregation and aggregation patterns between samples and highlighting the variation. All input variables were scaled to pareto scale and mean centered.

Molecular networks were generated for negative and positive ionization files applying Global Natural Products Social Molecular networking (GNPS, <http://gnps.ucsd.edu/ProteoSAFe/status/gnps-splash.jsp>) accessed date (14 April 2022). The parameters were adjusted for the network as follows: 0.02 Da parent mass tolerance, 0.01 Da fragment ion tolerance, 0.9 or above cosine score and a minimum of four matching peaks. Moreover, cytoscope open-source software (version 3.9.1) was used for network visualization.
