# Supplementary material for: Profiling of primary and phytonutrients in edible mahlab cherry (Prunus mahaleb L.) seeds in the context of its different cultivars and roasting as analyzed using molecular networking and chemometric tools
Source: PeerJ. 2023 Aug 30;11:e15908. doi: 10.7717/peerj.15908 (PMC10474835; doi:10.7717/peerj.15908)
Supplement: Supplemental Information 2 [file peerj-11-15908-s002.docx]

**Suppl. Table S1.** Metabolites identified in 100 % methanol extract of *Prunus mahaleb* L. seed cvs. using UPLC-ESI-MS in negative and positive ionization modes

| **No** | **Rt** | **Compound**  **name** | **Chemical class** | **[M-H]^-^/**  **[M+H]^+^** | **Formula** | **Error**  **ppm** | **MS/MS** | **EGM** | **WEM** | **REM** | **RREM** |
| --- | --- | --- | --- | --- | --- | --- | --- | --- | --- | --- | --- |
| 1 | 0.532 | Arginine | Amino acid | 175.1196 | C_6_H_15_N_4_O_2_^+^ | 3.69 | 158, 130 | + | + |  |  |
| 2 | 0.571 | Choline | *N*-Trimethylated amino acid | 104.1075 | C_5_H_15_NO^+^ | 6.46 | 60 | + | + | + | + |
| 3 | 0.585 | Sugar alcohol | Sugar | 181.0722 | C_6_H_13_O_6_^-^ | 2.11 | 59 | + | + |  | + |
| 4 | 0.629 | Sucrose | Sugar | 341.1091 | C_12_H_21_O_11_^-^ | 0.29 | 195, 75 | + | + |  | + |
| 5 | 0.638 | Gluconic acid | Organic acid | 195.051 | C_6_H_11_O_7_^-^ | 0.05 | 89, 71, 59 | + |  | + |  |
| 6 | 0.642 | Hexosyl-pentahydroxyheptanoic acid | Hydroxylated fatty acid | 387.1144 | C_13_H_23_O_13_^-^ | 0.36 | 341, 179, 119, 89 | + | + |  | + |
| 7 | 0.651 | Dihydroxy phenol galloyl hexoside | Polyphenol | 439.0857 | C_19_H_19_O_12_^-^ | 3.35 | 332, 96 |  |  | + | + |
| 8 | 0.662 | Malic acid | Organic acid | 133.0145 | C_4_H_5_O_5_^-^ | 0.88 | 96 |  | + | + | + |
| 9 | 0.76 | Unknown | -- | 112.087 | C_5_H_10_N_3_^+^ | 1.19 | 95, 78, 51 |  |  |  | + |
| 10 | 0.854 | Pyroglutamic acid | Amino acid | 130.05 | C_5_H_8_NO_3_^+^ | 1.43 | 57 | + | + | + | + |
| 11 | 0.855 | Nicotinic acid | Amino acid | 124.0393 | C_6_H_6_NO_2_^+^ | 2 | 80, 53 | + | + |  |  |
| 12 | 0.856 | Citric acid | Organic acid | 191.0197 | C_6_H_7_O_7_^-^ | 0.14 | 111, 87 | + | + | + | + |
| 13 | 0.86 | Lactic acid | Organic acid | 89.0244 | C_3_H_5_O_3_^-^ | 0.17 | 68 |  |  | + |  |
| 14 | 0.896 | Adenosine | Amino acid | 268.1041 | C_10_H_14_N_5_O_4_^+^ | 0.22 | 136, 57 | + | + | + | + |
| 15 | 0.9 | Tyrosine | Amino acid | 182.0811 | C_9_H_12_NO_3_^+^ | 0.13 | 91, 77 | + | + | + |  |
| 16 | 0.946 | Isoleucine | Amino acid | 132.1019 | C_6_H_14_NO_2_^+^ | 0.26 | 86, 69 | + | + | + | + |
| 17 | 0.976 | Glutaryl carnitine | Acyl carnitine | 276.1443 | C_12_H_22_NO_6_^+^ | 0.54 | 258, 230, 212, 86 |  | + | + |  |
| 18 | 0.978 | Succinic acid | Organic acid | 117.0194 | C_4_H_5_O_4_^-^ | 0.82 | 95, 73 |  |  | + | + |
| 19 | 1.002 | Deoxy fructosyl leucine | Amino acid derivative | 294.1547 | C_12_H_24_NO_7_^+^ | 0.2 | 276, 258, 230, 86 |  |  | + |  |
| 20 | 1.517 | Phenylalanine | Amino acid | 166.0865 | C_9_H_12_NO_2_^+^ | 1.46 | 120, 103, 51 | + | + | + | + |
| 21 | 1.567 | Unknown auxin derivative | Auxin | 368.0984 | C_16_H_18_NO_9_^-^ | 0.96 | 328, 257, 144, 59 | + | + |  |  |

**Table 1.** continued

| **No** | **Rt** | **Compound name** | **Chemical class** | **[M-H]^-^/**  **[M+H]^+^** | **Formula** | **Error**  **ppm** | **ms/ms** | **EGM** | **WEM** | **REM** | **RREM** |
| --- | --- | --- | --- | --- | --- | --- | --- | --- | --- | --- | --- |
| 22 | 1.616 | Unknown | -- | 146.0602 | C_9_H_8_NO^+^ | 0.89 | 128, 91, 51 | + | + |  |  |
| 23 | 1.709 | Unknown | -- | 358.1145 | C_15_H_20_NO_9_^-^ | 0.28 | 150, 71 | + | + |  |  |
| 24 | 1.715 | Unknown | -- | 134.0603 | C_8_H_8_NO^+^ | 1.7 | 106, 79, 51 | + | + | + |  |
| 25 | 1.747 | Unknown | -- | 649.2209 | C_24_H_41_O_20_^+^ | 3.29 | 336, 174 | + |  |  |  |
| 26 | 1.991 | Unknown | -- | 297.1558 | C_12_H_25_O_8_^+^ | 0.14 | 192, 132, 88 |  |  |  | + |
| 27 | 2.228 | Unknown | -- | 188.0711 | C_11_H_10_NO_2_^+^ | 1.28 | 146, 74 | + | + | + |  |
| 28 | 2.407 | Coumaric acid-*O*- dihexoside | Hydroxycinnamtes | 487.1454 | C_21_H_27_O_13_^-^ | 0.91 | 256, 163, 119 | + | + |  |  |
| 29 | 2.435 | Unknown amino sugar | Amino acid derivative | 506.1867 | C_21_H_32_NO_13_^+^ | 0.18 | 289,165, 85 | + | + |  |  |
| 30 | 2.475 | Unknown | -- | 582.2395 | C_24_H_40_NO_15_^+^ | 0.15 | 448, 297, 107 |  |  | **+** |  |
| 31 | 2.526 | Unknown nitrogenous compound | Nitrogenous compound | 102.0914 | C_5_H_12_NO^+^ | 0 | 57 | + |  |  |  |
| 32 | 2.618 | Unknown | -- | 146.1174 | C_7_H_16_NO_2_^+^ | 0.03 | 57 | + |  |  |  |
| 33 | 2.658 | Unknown | -- | 447.15 | C_19_H_27_O_12_^-^ | 2.44 | 299, 192, 133 |  |  | **+** |  |
| 34 | 2.665 | Unknown | -- | 420.1863 | C_18_H_30_NO_10_^+^ | 0.36 | 343, 223, 107 |  |  | **+** |  |
| 35 | 2.717 | Unknown | -- | 450.1972 | C_19_H_32_NO_11_^+^ | 0.76 | 217, 91 | + | + | **+** | **+** |
| 36 | 2.81 | Unknown | -- | 651.1844 | C_37_H_31_O_11_^-^ | 0.35 | 573, 423, 325, 163, 119 | + |  |  | **+** |
| 37 | 2.824 | Tyrosinyl hexoside. | Amino acid derivative | 344.1215 | C_15_H_22_NO_8_^+^ | 0.76 | 165, 147 | + | + | **+** | **+** |
| 38 | 2.847 | *p*-Coumaric acid | Hydroxycinnamtes | 165.0548 | C_9_H_9_O_3_^+^ | 1.25 | 119 | + | + |  |  |
| 39 | 2.855 | *p*-Coumaric acid-*O*-hexoside | Hydroxycinnamtes | 325.0785 | C_15_H_17_O_8_^-^ | 2.95 | 277, 163, 119, 59 | + | + | **+** | **+** |
| 40 | 2.86 | Phenylacetaldehyde | Phenyl propane aldehyde | 119.0509 | C_8_H_7_O^-^ | 5.52 | 59 | + |  | **+** |  |

**Table 1.** continued

| **No** | **Rt** | **Compound name** | **Chemical class** | **[M-H]^-^/**  **[M+H]^+^** | **Formula** | **Error**  **ppm** | **ms/ms** | **EGM** | **WEM** | **REM** | **RREM** |
| --- | --- | --- | --- | --- | --- | --- | --- | --- | --- | --- | --- |
| 41 | 2.943 | Unknown | -- | 489.1525 | C_28_H_25_O_8_^-^ | 1.33 | 165, 121 | + | + |  |  |
| 42 | 3.031 | Methoxy hydroxy amygdalin | Cyanogenic glycoside | 502.1559 | C_21_H_28_NO_13_^-^ | 1.62 | 456, 325, 221, 178, 59 | + | + | **+** | **+** |
| 43 | 3.04 | Unknown | -- | 475.1923 | C_20_H_31_N_2_O_11_^+^ | 0.02 | 145 | + | + | **+** | **+** |
| 44 | 3.047 | Amygdalin | Cyanogenic glycoside | 456.1505 | C_20_H_26_NO_11_^-^ | 1.62 | 323, 263, 221 |  |  | **+** | **+** |
| 45 | 3.237 | Unknown | -- | 519.1712 | C_22_H_31_O_14_^-^ | 0.66 | 195, 151, 59 |  | + |  |  |
| 46 | 3.29 | Norharman | *β*-Carboline | 169.076 | C_11_H_7_N_2_^-^ | 0.05 | 115 |  |  |  | **+** |
| 47 | 3.329 | Unknown | -- | 230.1177 | C_14_H_16_NO_2_^+^ | 0.43 | 91 |  |  | **+** | **+** |
| 48 | 3.342 | Ferulic acid-*O*-hexoside dimer | Hydroxycinnamtes | 711.2147 | C_32_H_39_O_18_^-^ | 0.38 | 355, 193, 59 | + | + | **+** |  |
| 49 | 3.401 | Vanillin | Phenolic aldehyde | 153.0548 | C_8_H_9_O_3_^+^ | 1.17 | 125 | + | + |  |  |
| 50 | 3.496 | Hydroxybenzaldehyde | Phenyl propane | 121.0294 | C_7_H_5_O_2_^-^ | 1.22 | 92, 77 |  |  | **+** | **+** |
| 51 | 3.532 | Dihydrocoumaroyl-*O*- hexoside (Dihydromelilotoside) | Hydroxycinnamtes | 327.1082 | C_15_H_19_O_8_^-^ | 1.08 | 259, 165, 121 | + | + |  |  |
| 52 | 3.536 | Unknown amino sugar | Amino sugar | 346.1498 | C_15_H_24_NO_8_^+^ | -0.41 | 215, 149 | + | + |  |  |
| 53 | 3.541 | Dihydrocoumaroyl-*O*- hexoside dimer | Hydroxycinnamtes | 655.2164 | C_30_H_39_O_16_^-^ | 0.82 | 327, 165, 121 | + | + |  |  |
| 54 | 3.687 | Unknown | -- | 340.1034 | C_15_H_18_NO_8_^-^ | 1.23 | 284, 71 |  |  |  | **+** |
| 55 | 3.86 | Hexosyl methoxyphenyl propanoic acid | Hydroxycinnamtes | 357.1186 | C_16_H_21_O_9_^-^ | 1.63 | 195, 151 | + | + |  |  |
| 56 | 3.876 | Dihydroferulic acid-*O*- hexoside dimer | Hydroxycinnamtes | 715.2462 | C_32_H_43_O_18_^-^ | 0.13 | 621, 357, 195 | + | + |  |  |
| 57 | 3.884 | Unknown | -- | 376.1603 | C_16_H_26_NO_9_^+^ | 0.01 | 179, 55 | + | + |  |  |
| 58 | 4.263 | Ferulic acid-*O*-hexoside | Hydroxycinnamtes | 355.1033 | C_16_H_19_O_9_^-^ | 0.63 | 193,134, 59 | + | + | **+** | **+** |

**Table 1.** continued

| **No** | **Rt** | **Compound name** | **Chemical class** | **[M-H]^-^/**  **[M+H]^+^** | **Formula** | **Error**  **ppm** | **ms/ms** | **EGM** | **WEM** | **REM** | **RREM** |
| --- | --- | --- | --- | --- | --- | --- | --- | --- | --- | --- | --- |
| 59 | 4.29 | Ferulic acid | Hydroxycinnamtes | 195.0652 | C_10_H_11_O_4_^+^ | 0.08 | 177 | + | + | **+** | **+** |
| 60 | 4.411 | Unknown | -- | 573.9756 | C_22_H_8_NO_13_^-^ | 1.8 | 522, 412, 161 | + |  |  |  |
| 61 | 4.423 | Unknown | -- | 700.0299 | C_36_H_15_NO_13_P^-^ | 1.59 | 692, 539, 161 |  |  |  | **+** |
| 62 | 4.483 | Apigenin-*C*-hexoside | Flavonoid | 431.0972  433.1128 | C_21_H_19_O_10_^-^  C_21_H_21_O_10_^+^ | 1.31  0.44 | 341, 311, 283, 269  343, 313, 283, 271 |  |  | **+** |  |
| 63 | 4.648 | Prupersin E | Cyanogenic glycoside | 563.1878 | C_26_H_31_N_2_O_12_^+^ | 0.84 | 430, 268, 124 |  |  | **+** | **+** |
| 64 | 4.726 | Flavone (luteolin)-methoxy-*C*-hexoside | Flavonoid | 461.1087  463.1234 | C_22_H_21_O_11_^-^  C_22_H_23_O_11_^+^ | 1.26  0.37 | 371, 341, 298  373, 343 |  |  | **+** |  |
| 65 | 4.922 | Caffeyl alcohol | Hydroxycinnamtes | 165.0557 | C_9_H_9_O_3_^-^ | 0.18 | 147, 121 |  | + |  |  |
| 66 | 5.226 | Dihydroferulic acid | Hydroxycinnamtes | 195.006 | C_10_H_11_O_4_^-^ | 0.16 | 136,121 | + | + |  |  |
| 67 | 5.424 | Unknown | -- | 187.0975 | C_9_H_15_O_4_^-^ | 0.32 | 212 | + | + | **+** |  |
| 68 | 5.858 | Unknown | -- | 739.3533 | C_37_H_55_O_15_^-^ | 1.93 | 605, 513, 321 |  |  | **+** |  |
| 69 | 5.92 | Coumarin | Coumarin | 147.0442 | C_9_H_7_O_2_^+^ | 0.8 | 132, 119, 103, 91 | + | + | **+** | **+** |
| 70 | 6.39 | Unknown | -- | 497.2899 | C_30_H_41_O_6_^+^ | 0.43 | 482, 349, 209, 104 |  |  | **+** |  |
| 71 | 6.41 | Unknown | -- | 721.3436 | C_37_H_53_O_14_^-^ | 0.99 | 675, 596, 495, 321 |  |  | **+** |  |
| 72 | 6.44 | Unknown | -- | 499.3056 | C_30_H_43_O_6_^+^ | 0.24 | 481, 317, 167, 113 |  |  | **+** |  |
| 73 | 6.96 | Methoxy coumarin (herniarin) | Coumarin | 177.0548 | C_10_H_9_O_3_^+^ | 2.67 | 162, 133, 121, 85 | + | + | **+** | **+** |
| 74 | 7.981 | Unknown | -- | 170.1542 | C_10_H_20_NO^+^ | 1.54 | 142, 112, 86, 57 | + |  |  |  |
| 75 | 8.077 | Unknown | -- | 736.3905 | C_38_H_58_NO_13_^+^ | -0.8 | 341, 161, 74 |  | + |  |  |
| 76 | 8.284 | Unknown | -- | 738.1059 | C_38_H_60_NO_13_^+^ | 0.5 | 335, 241, 113 |  | + |  |  |
| 77 | 8.561 | Unknown | -- | 376.1755 | C_20_H_26_NO_6_^+^ | 0.04 | 253, 91 | + | + |  |  |
| 78 | 9.204 | Nonamide | Fatty acyl amide | 158.1543 | C_9_H_20_NO^+^ | 2.13 | 57 | + | + | **+** | **+** |
| 79 | 9.57 | Decanamide | Fatty acyl amide | 172.1695 | C_10_H_22_NO^+^ | 0.51 | 121, 116, 74, 60 |  |  | **+** |  |

**Table 1.** continued

| **No** | **Rt** | **Compound name** | **Chemical class** | **[M-H]^-^/**  **[M+H]^+^** | **Formula** | **Error**  **ppm** | **ms/ms** | **EGM** | **WEM** | **REM** | **RREM** |
| --- | --- | --- | --- | --- | --- | --- | --- | --- | --- | --- | --- |
| 80 | 10.657 | Unknown | -- | 309.2067 | C_18_H_29_O_4_^-^ | 1.59 | 291, 209, 99 |  | + |  |  |
| 81 | 10.68 | Oxo-octadecatrienoic acid | Fatty acid | 293.2117 | C_18_H_29_O_3_^+^ | 1.39 | 275, 237, 197, 137 | + | + |  |  |
| 82 | 10.886 | Unknown | -- | 286.1441 | C_17_H_18_NO_3_^-^ | 0.99 | 201, 135, 69 |  | + |  |  |
| 83 | 11.414 | Unknown | -- | 574.338 | C_32_H_48_NO_8_^+^ | 1.1 | 497, 385, 219, 111 |  |  | **+** |  |
| 84 | 11.565 | Benzyl tetradecanamine | Arylalkylamine | 304.3001 | C_21_H_38_N^+^ | 0.71 | 212, 91 | + | + | **+** | **+** |
| 85 | 11.747 | Unknown | -- | 415.2117 | C_24_H_31_O_6_^+^ | 0.49 | 296, 119 | + | + | **+** | **+** |
| 86 | 11.824 | Unknown oxylipid | Oxylipid | 307.1913 | C_18_H_27_O_4_^-^ | 0.76 | 223, 137, 99 |  | + |  |  |
| 87 | 12.898 | Nonadecanamine | Amino lipid | 284.3311 | C_19_H_42_N^+^ | 0.42 | 202, 60 | + | + | **+** | **+** |
| 88 | 12.931 | Unknown | -- | 476.2773 | C_30_H_40_NO_4_^+^ | 2.15 | 279, 196 |  | + |  |  |
| 89 | 12.972 | Benzyl hexadecanamine | Arylalkylamine | 332.3314 | C_23_H_42_N^+^ | 0.59 | 240, 91 | + | + | **+** | **+** |
| 90 | 13.11 | Unknown | -- | 520.3404 | C_28_H_46_N_3_O_6_^+^ | 1.42 | 258, 184 |  | + | **+** | **+** |
| 91 | 13.129 | Unknown sphingolipid | Shingolipid | 564.3296 | C_27_H_51_NO_9_P^-^ | 2.24 | 279 |  | + | **+** | **+** |
| 92 | 13.78 | Unknown | -- | 496.3402 | C_26_H_46_N_3_O_6_^+^ | 1.57 | 419, 258, 184 |  | + | **+** | **+** |
| 93 | 14.22 | Unknown sphingolipid | Shingolipid | 522.356 | C_26_H_51_NO_7_P^-^ | 1.07 | 279, 196 |  |  | **+** | **+** |
| 94 | 14.288 | Heptanamine | Amino lipid | 312.3626 | C_21_H_46_N^+^ | 0.27 | 224, 60 | + | + |  |  |
| 95 | 14.304 | Unknown sphingolipid | Shingolipid | 566.3453 | C_27_H_53_NO_9_P^-^ | 2.08 | 281, 78 |  | + | **+** | **+** |
| 96 | 14.371 | Stearidonic acid | Fatty acid | 277.2164 | C_18_H_29_O_2_^+^ | 0.48 | 133 | + | + |  |  |
| 97 | 14.31 | Benzyl  octadecylamine | Arylalkylamine | 360.2636 | C_25_H_46_N^+^ | 0.04 | 268, 91 | + | + | **+** | **+** |
| 98 | 14.321 | Unknown | -- | 595.2878 | C_34_H_43_O_9_^-^ | 5.85 | 456, 265,115 |  | + | **+** |  |
| 99 | 14.619 | Hydroxylinoleic acid | Fatty acid | 295.2268 | C_18_H_31_O_3_^+^ | 0.26 | 277, 195 | + |  |  |  |
| 100 | 15.088 | Benzofurandione | Benzofuran | 149.0237 | C_8_H_5_O_3_^+^ | -2.2 | 57 |  | + | **+** |  |

**Table 1.** continued

| **No** | **Rt** | **Compound name** | **Chemical class** | **[M-H]^-^/**  **[M+H]^+^** | **Formula** | **Error**  **ppm** | **ms/ms** | **EGM** | **WEM** | **REM** | **RREM** |
| --- | --- | --- | --- | --- | --- | --- | --- | --- | --- | --- | --- |
| 101 | 15.795 | Linaloyl ethanolamide | Fatty ethanolamide | 324.2898 | C_20_H_38_NO_2_^+^ | 0.04 | 306, 62 | + | + |  |  |
| 102 | 16.606 | Palmitoyl ethanolamide | Fatty ethanolamide | 300.2898 | C_18_H_38_NO_2_^+^ | -0.16 | 283, 62 | + |  |  |  |
| 103 | 17.184 | Oleoyl ethanolamide | Fatty ethanolamide | 326.3055 | C_30_H_40_NO_2_^+^ | -0.31 | 309, 62 | + |  |  |  |
| 104 | 17.684 | Unknown oxylipid | Oxylipid | 377.1422 | C_23_H_21_O_5_^-^ | -6.35 | 217, 99 |  | + |  |  |
| 105 | 17.699 | Octadecenedioic acid | Fatty acid | 311.2229 | C_18_H_31_O_4_^-^ | 0.05 | 293, 171 |  | + |  |  |
| 106 | 18.277 | Unknown oxylipid | Oxylipid | 379.1581 | C_23_H_23_O_5_^-^ | -6.89 | 230, 99 |  | + | **+** |  |
| 107 | 18.295 | Unknown | -- | 325.2381 | C_19_H_33_O_4_^-^ | 1.15 | 307, 225, 113 |  | + |  |  |
| 108 | 19.518 | Unknown oxylipid | Oxylipid | 381.1731 | C_23_H_25_O_5_^-^ | -5.56 | 289, 198, 99 |  | + |  |  |
| 109 | 19.541 | Unknown steroid | Steroid | 327.2539 | C_30_H_53_O_7_^-^ | 1.39 | 410, 281 | + | + |  |  |
| 110 | 21.268 | Docosenamide | Fatty acyl amide | 338.3413 | C_22_H_44_NO^+^ | 0.17 | 321, 303, 233, 57 | + | + | **+** |  |
