# Supplementary figures and images for: Profiling of primary and phytonutrients in edible mahlab cherry (Prunus mahaleb L.) seeds in the context of its different cultivars and roasting as analyzed using molecular networking and chemometric tools
Source: PeerJ. 2023 Aug 30;11:e15908. doi: 10.7717/peerj.15908 (PMC10474835; doi:10.7717/peerj.15908)

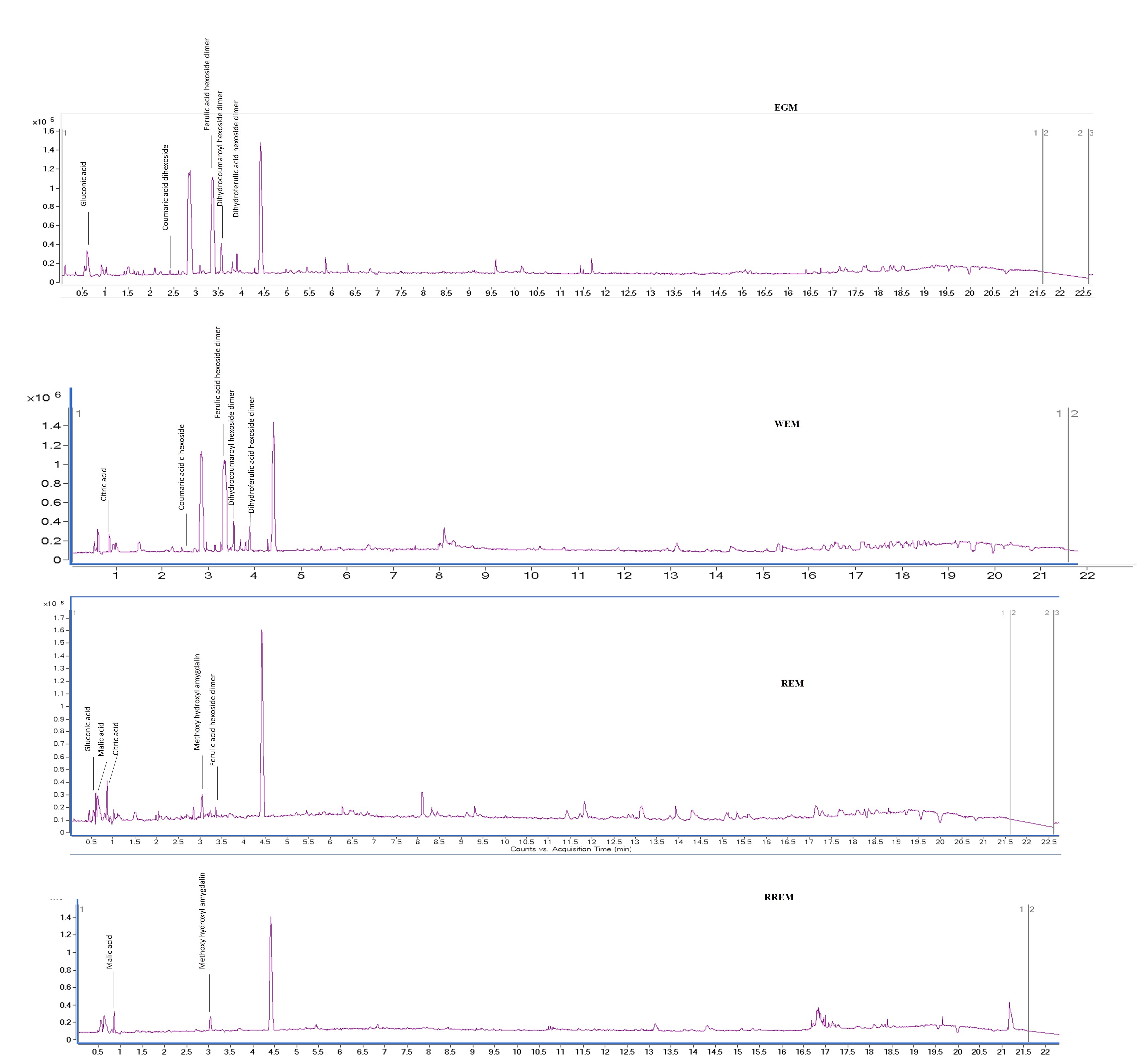

Supplement: Supplemental Information 3 [file peerj-11-15908-s003.jpg]

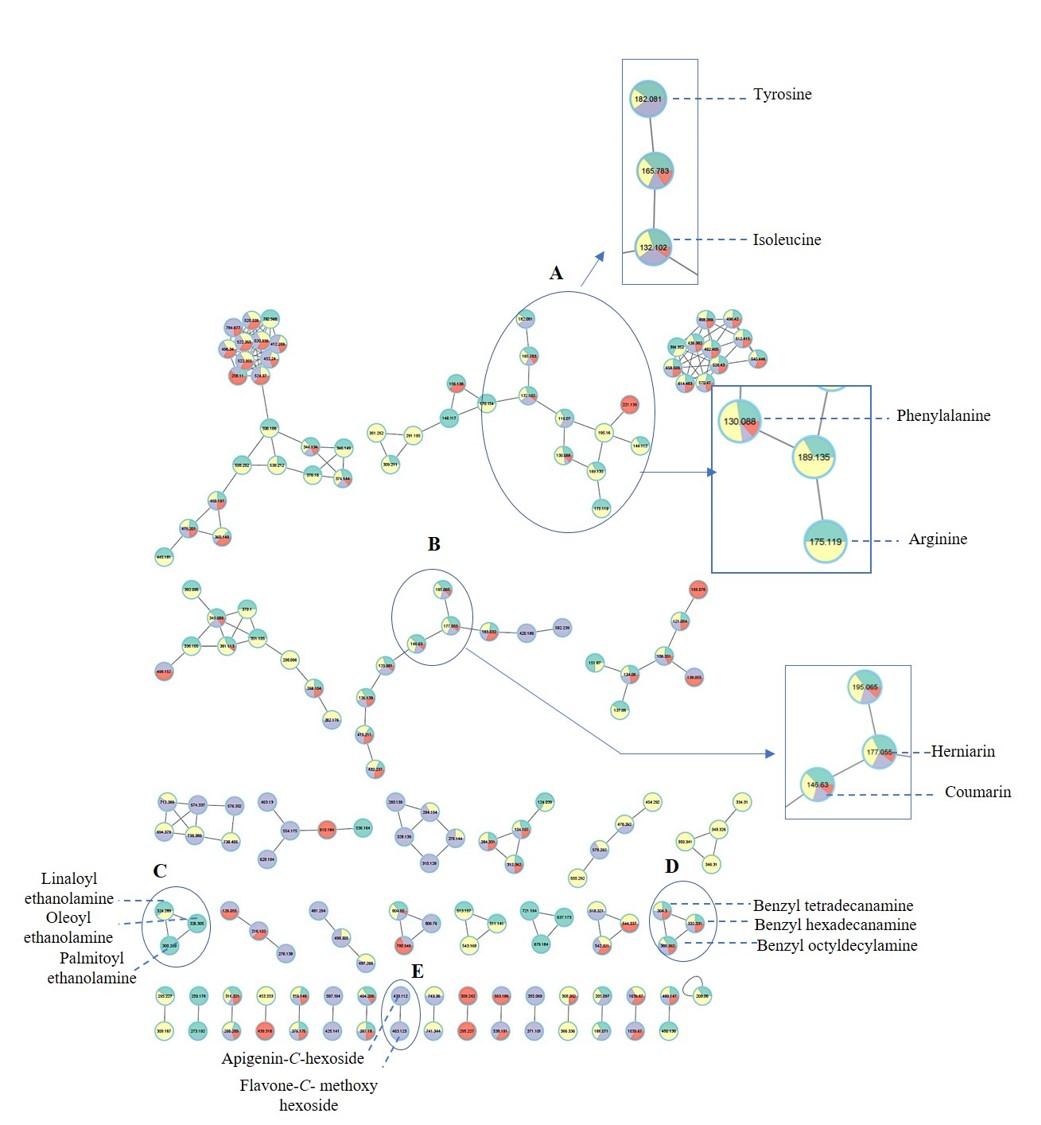

Supplement: Supplemental Information 4 — A: amino acids, cluster B: coumarins, cluster C: fatty acid ethanolamines, cluster D: arylalkylamines and cluster E: flavonoids. [file peerj-11-15908-s004.jpeg]

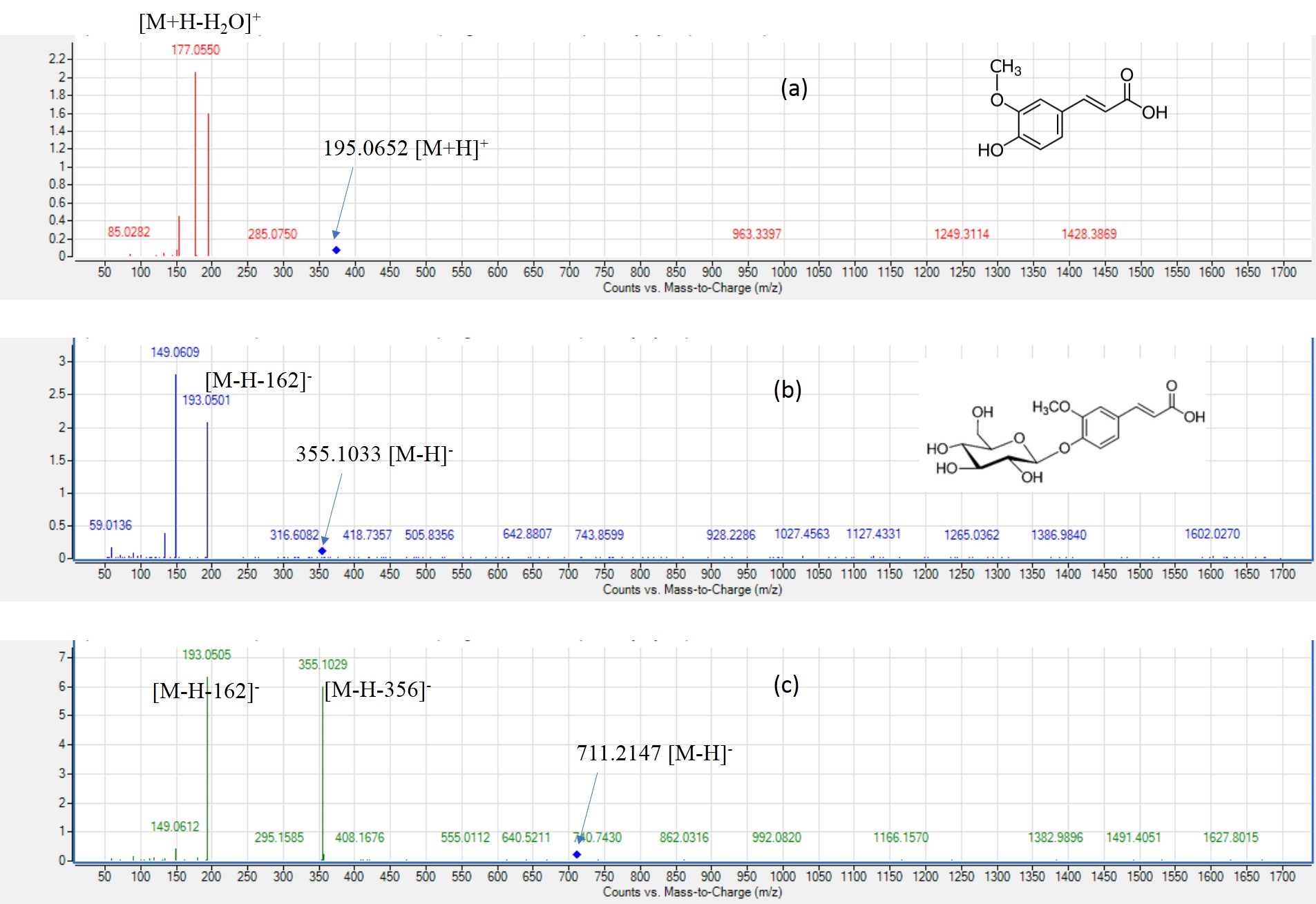

Supplement: Supplemental Information 5 — (a) ferulic acid (peak 59) [M+H]+ m/z 195.0652, (b) ferulic acid-O-hexoside (peak 58) [M-H]- m/z 355.1033, C16H28NO20O28NO9-, (c) ferulic acid-O-hexoside dimer (peak 48) [M-H]- m/z 711.2147, C28NO32H28NO40O28NO18-. [file peerj-11-15908-s005.jpg]

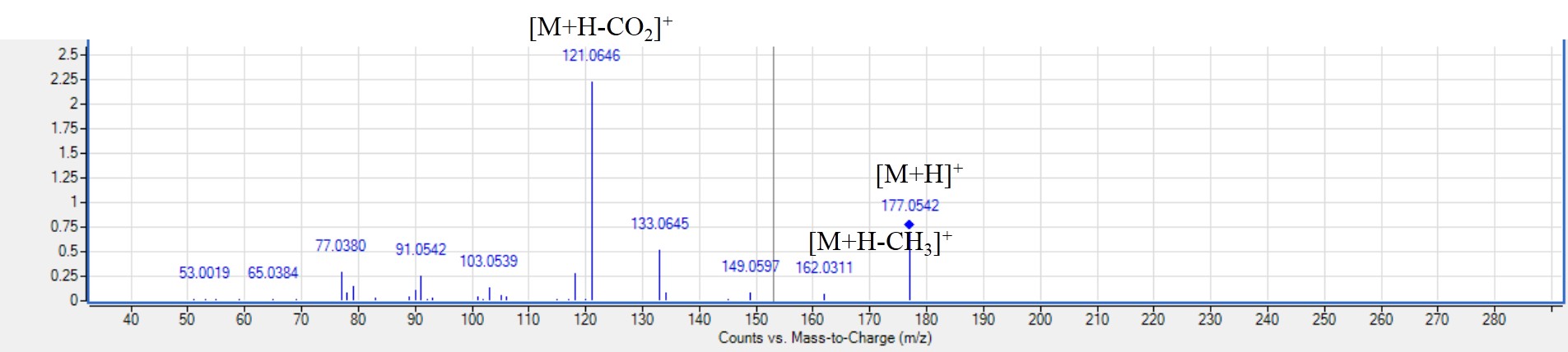

Supplement: Supplemental Information 6 [file peerj-11-15908-s006.jpg]

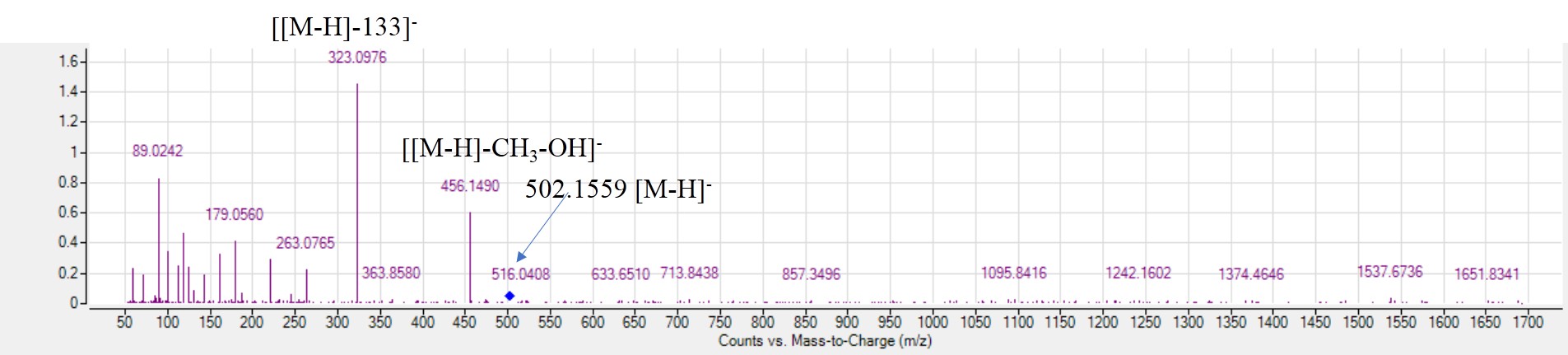

Supplement: Supplemental Information 7 [file peerj-11-15908-s007.jpg]

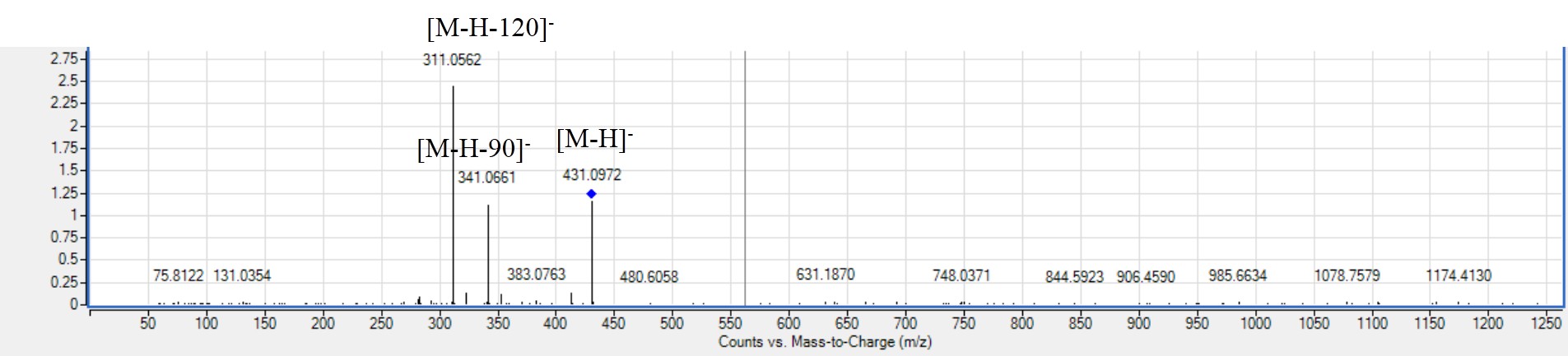

Supplement: Supplemental Information 8 [file peerj-11-15908-s008.jpg]

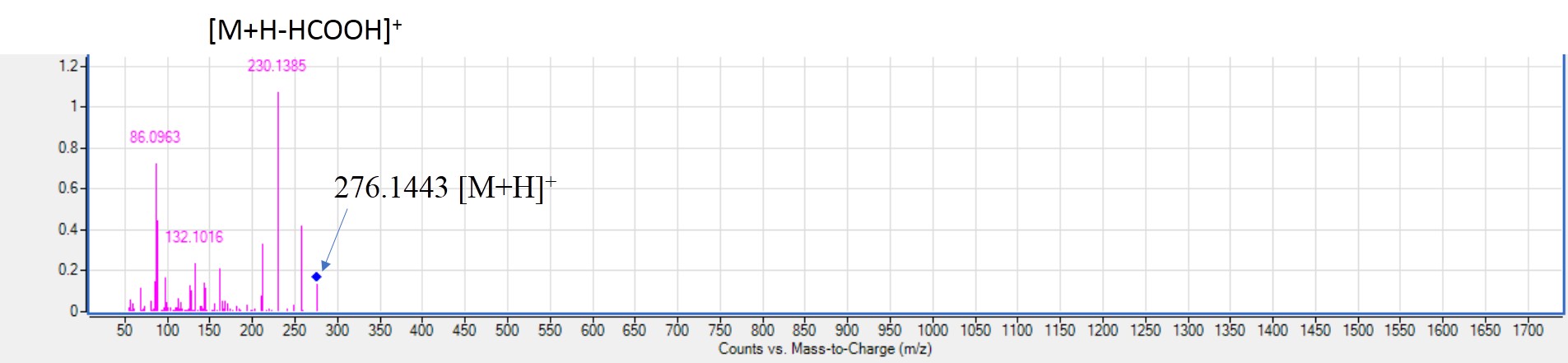

Supplement: Supplemental Information 9 [file peerj-11-15908-s009.jpg]

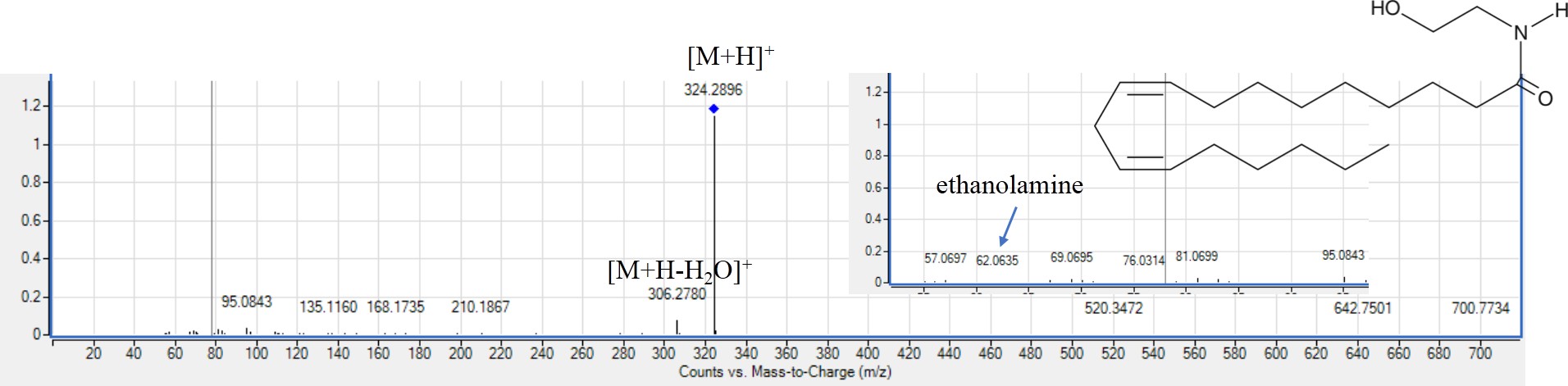

Supplement: Supplemental Information 10 [file peerj-11-15908-s010.jpg]

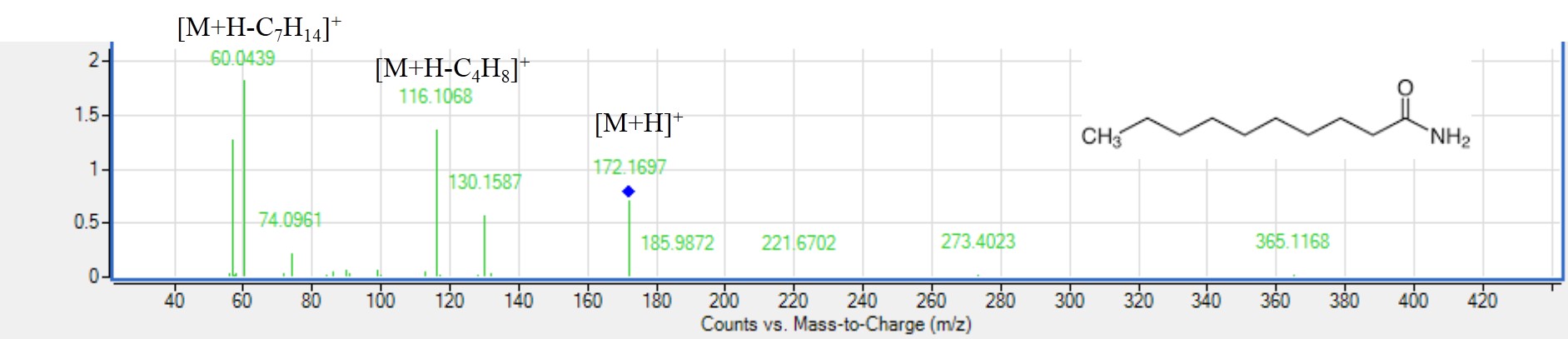

Supplement: Supplemental Information 11 [file peerj-11-15908-s011.jpg]

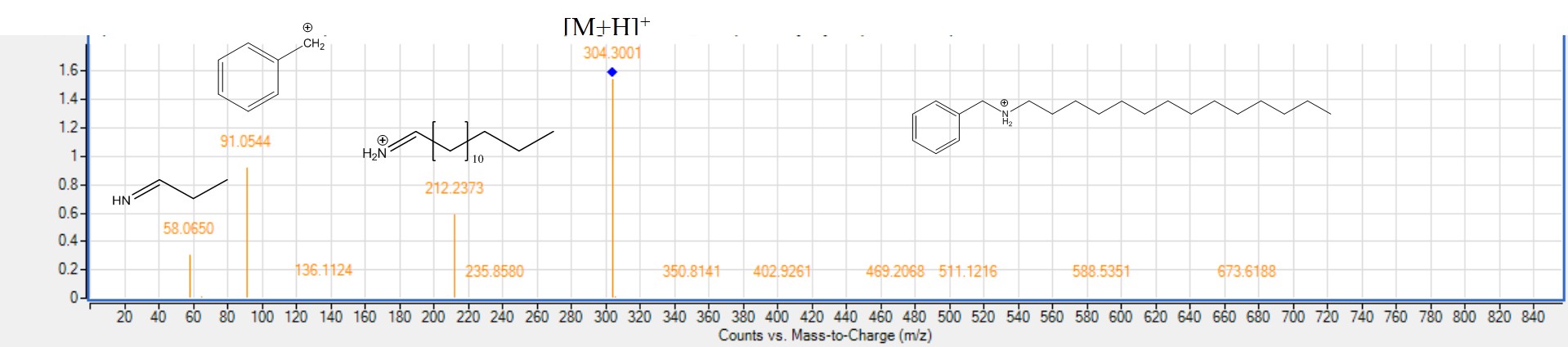

Supplement: Supplemental Information 12 [file peerj-11-15908-s012.jpg]

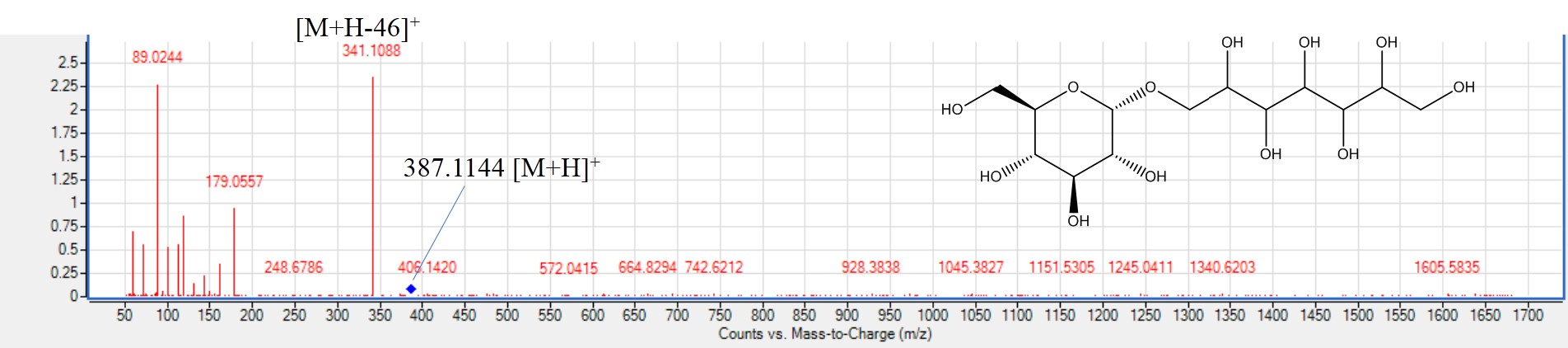

Supplement: Supplemental Information 13 [file peerj-11-15908-s013.jpg]
